# Dichloridobis[ethyl 2-(2-amino-1,3-thia­zol-4-yl)acetate-κ^2^ *O*,*N* ^3^]cadmium

**DOI:** 10.1107/S1600536812021976

**Published:** 2012-05-26

**Authors:** Lai-Jun Zhang, Fa-Yun Chen, Guang-Yi Liu, Xiao Chen, Zhi-Feng Chen

**Affiliations:** a School of Chemistry and Chemical Engineering, Shangrao Normal University, Shangrao 334001, People’s Republic of China

## Abstract

The asymmetric unit of the title compound, [CdCl_2_(C_7_H_10_N_2_O_2_S)_2_], contains two complex molecules with similar configurations. The Cd^II^ atoms are each six-coordinated by two thiazole N and two carbonyl O atoms from the 2-(2-amino-1,3-thiazol-4-yl)acetate ligand, and by two Cl^−^ anions in a distorted octa­hedral geometry. In the crystal, intra- and inter­molecular N—H⋯Cl hydrogen bonds create parallel chains along [1-10]. C—H⋯Cl inter­actions also occur.

## Related literature

For the pharmacological activity, including anti­tumor activity, of metal complexes with thia­zole ligands, see: Alexandru *et al.* (2010 ▶); Chang *et al.* (1982 ▶). For related structures and preparative procedures, see: Alexandru *et al.* (2010 ▶); He *et al.* (2009 ▶); Siddiqui *et al.* (2009 ▶); Yang *et al.* (2009 ▶); Usman *et al.* (2003 ▶); Zhang *et al.* (2008*a* ▶,*b* ▶, 2009 ▶).
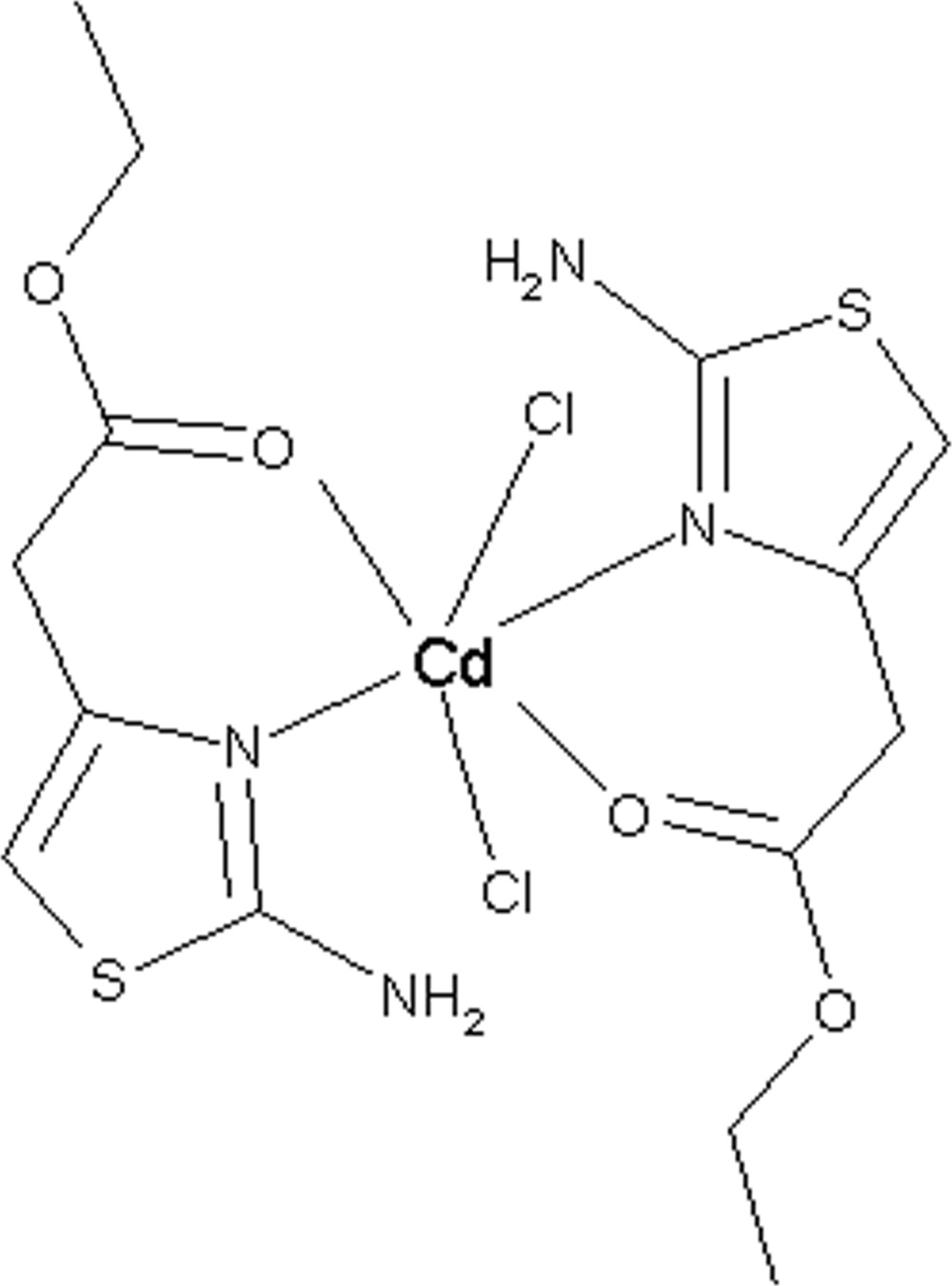


## Experimental

### 

#### Crystal data

[CdCl_2_(C_7_H_10_N_2_O_2_S)_2_]
*M* *_r_* = 555.76
Monoclinic, 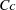

*a* = 16.860 (3) Å
*b* = 16.630 (3) Å
*c* = 16.220 (3) Å
β = 105.41 (3)°
*V* = 4384.3 (15) Å^3^
*Z* = 8
Mo *K*α radiation
μ = 1.46 mm^−1^
*T* = 293 K
0.13 × 0.11 × 0.08 mm

#### Data collection

Bruker APEXII CCD area-detector diffractometer
Absorption correction: multi-scan (*SADABS*; Sheldrick, 1996 ▶) *T*_min_ = 0.833, *T*_max_ = 0.892
14070 measured reflections
8707 independent reflections
8223 reflections with *I* > 2σ(*I*)
*R*_int_ = 0.015

#### Refinement

*R*[*F* ^2^ > 2σ(*F* ^2^)] = 0.022
*wR*(*F* ^2^) = 0.048
*S* = 1.02
8707 reflections
492 parameters
2 restraints
H-atom parameters constrained
Δρ_max_ = 0.48 e Å^−3^
Δρ_min_ = −0.28 e Å^−3^
Absolute structure: Flack (1983 ▶), 3396 Friedel pairs
Flack parameter: 0.003 (12)

### 

Data collection: *APEX2* (Bruker, 2007 ▶); cell refinement: *SAINT-Plus* (Bruker, 2007 ▶); data reduction: *SAINT-Plus*; program(s) used to solve structure: *SHELXS97* (Sheldrick, 2008 ▶); program(s) used to refine structure: *SHELXL97* (Sheldrick, 2008 ▶); molecular graphics: *SHELXTL* (Sheldrick, 2008 ▶); software used to prepare material for publication: *SHELXTL*.

## Supplementary Material

Crystal structure: contains datablock(s) I, global. DOI: 10.1107/S1600536812021976/vn2035sup1.cif

Structure factors: contains datablock(s) I. DOI: 10.1107/S1600536812021976/vn2035Isup2.hkl

Additional supplementary materials:  crystallographic information; 3D view; checkCIF report

## Figures and Tables

**Table 1:** Selected bond lengths (Å)

Cd1—N3	2.343 (2)
Cd1—N4	2.315 (2)
Cd1—O7	2.475 (2)
Cd1—O8	2.384 (2)
Cd1—Cl3	2.5041 (8)
Cd1—Cl4	2.5664 (8)
Cd2—N1	2.344 (2)
Cd2—N2	2.347 (2)
Cd2—O4	2.511 (2)
Cd2—O6	2.377 (3)
Cd2—Cl1	2.5491 (9)
Cd2—Cl2	2.5051 (12)

**Table 2:** Hydrogen-bond geometry (Å, °)

*D*—H⋯*A*	*D*—H	H⋯*A*	*D*⋯*A*	*D*—H⋯*A*
N5—H5*A*⋯Cl3	0.86	2.46	3.291 (3)	163
N6—H6*A*⋯Cl4	0.86	2.43	3.277 (3)	167
N7—H7*A*⋯Cl1	0.86	2.43	3.242 (3)	157
N8—H8*A*⋯Cl2	0.86	2.43	3.248 (3)	160
C14—H14*B*⋯Cl2	0.97	2.80	3.607 (3)	141
C34—H34*A*⋯Cl2	0.93	2.81	3.687 (4)	158
N5—H5*B*⋯Cl1^i^	0.86	2.67	3.435 (3)	149
N6—H6*B*⋯Cl1^ii^	0.86	2.41	3.189 (3)	152
N8—H8*B*⋯Cl4^iii^	0.86	2.57	3.373 (3)	155
C35—H35*A*⋯Cl3^iv^	0.93	2.82	3.681 (4)	153

Symmetry codes: (i) 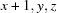; (ii) 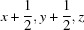; (iii) 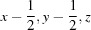; (iv) 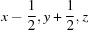.

**Fig. 1.**
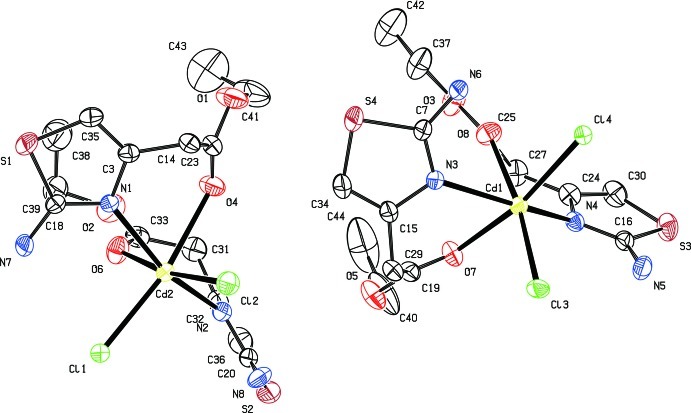
The molecular structure of the title complex. Displacement ellipsoids are drawn at the 30% probability level. All hydrogen atoms were omitted for clarity.

**Fig. 2.**
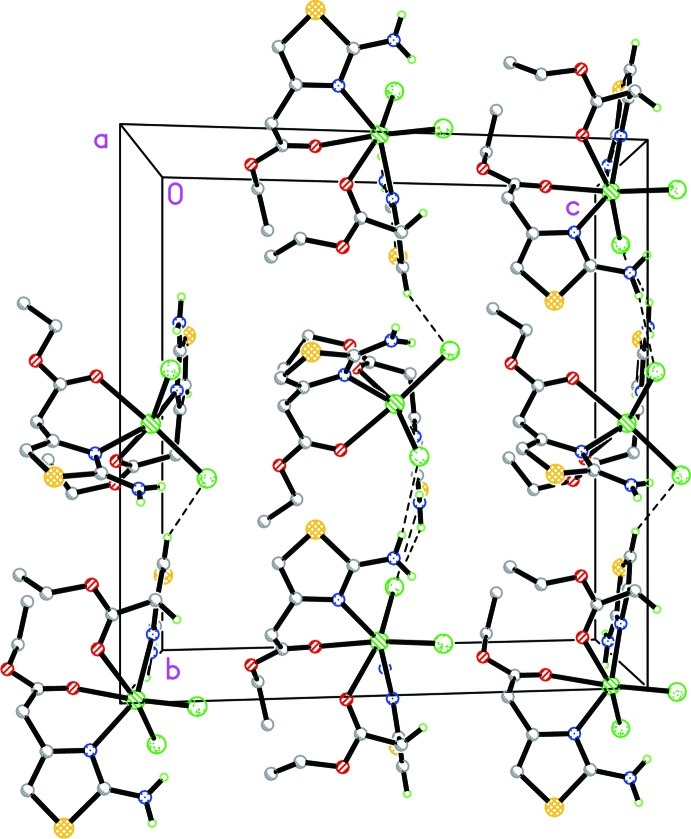
The crystal packing of the title compound viewed along the a axis. Intermolecular and intramolecular N—H···Cl and C—H···Cl hydrogen bonds are indicated by dashed lines. All hydrogen atoms not involved in hydrogen bonding were omitted for clarity.

## supplementary crystallographic information

### Comment

Metal complexes with thiazole ligands have received attention as potential metal-based drugs due to pharmacological activity (Chang *et al.*, 1982). Organic ligands containing aminothiazole group such as ethyl 2-aminothiazole-4-acetate (EATA) and 2-amino-4-thiazole acetate (ATA) possess strong coordination ability and display diverse coordination modes due to the present of N, O coordination atoms (Usman *et al.*, 2003). We have recently determined the crystal structures of metal complexes with EATA or ATA as ligands, including bicoordinated [Ag(C_7_H_10_N_2_O_2_S)_2_]NO_3_ (Zhang *et al.*, 2008*a*), four-coordinated 2-amino-4-thiazole acetic acid (ATAA) (Zhang *et al.*, 2008*b*), five-coordinated [Zn(C_5_H_5_N_2_O_2_S)_2_(H_2_O)] (Zhang *et al.*, 2009). Single crystal structure determinations of five-coordinated Ni^II^ (He *et al.*, 2009), Mn^II^ (Alexandru *et al.*, 2010) and Zn^II^ (Siddiqui *et al.*, 2009; Yang *et al.*, 2009) complexes with aminothiazole acetate (ATA) derivative have been carried out by other groups, and it is found that the corresponding Cu^II^ and Mn^II^ complexes exhibit promising antitumor activity against cancerous cells (HeLa) (Alexandru *et al.*, 2010). Here, we report a new six-coordinated title complex [Cd(C_7_H_10_N_2_O_2_S)_2_Cl_2_], I, using EATA as ligand. Interestingly, when cadmium chloride hydrate instead of ZnSO_4_ was used as starting material, EATA molecule would not hydrolyze under ultrasonic irradiation at room temperature.

In the crystal structure, the central Cd atom is six-coordinated by two N atoms, two O atoms and two Cl atoms in a distorted octahedral geometry, each EATA ligand provides one thiazole nitrogen and one oxygen atoms as coordinated atoms (Table 1, Fig. 1). Numerous hydrogen bonds including the intramolecular N—H···Cl hydrogen bonds between one H atom of NH_2_ group on a thiazole ring and one Cl atom from the same molecule, the intermolecular N—H···Cl hydrogen bonds between the other H atom of NH_2_ group on the thiazole ring and Cl atom from another molecule, and the intermolecular and intramolecular C—H···Cl hydrogen bonds between the H atom of the thiazole ring or CH_2_ group and Cl atom are formed, which further stabilize and aggregate the [Cd(C_7_H_10_N_2_O_2_S)_2_Cl_2_] molecules and create parallel one-dimensional chains along [1-10] (Table 2, Fig. 2).

### Experimental

An ethanol-water solution of ethyl 2-aminothiazole-4-acetate (EATA) was prepared by first dissolving EATA (1 mmol, 0.186 g) in ethanol (5 ml) and then adding distilled water (5 ml) under stirring. Then, CdCl_2_ (1 mmol, 0.228 g) was added and dissolved after a 10-minutes ultrasonic treatment. The resulting solution was filtered and left at room temperature for overnight. Big block pale-yellow single crystals were obtained in about 38% yield (based on Cd).

### Refinement

All hydrogen atoms have been refined in a riding mode model on their carrier atom, with C–H = 0.93 Å (thiazole ring), 0.96 Å (CH_2 _group), and 0.97 Å (CH_3 _group), N–H = 0.86 Å and *U*iso(H) = 1.2*U*eq(N), *U*iso(H) = 1.2*U*eq (C from thiazole ring and CH_2_ group), *U*iso(H) = 1.5*U*eq (C from CH_3_ group). The methyl groups appear to be slightly disordered, but attempts to model this disorder did not result in a better fit.

### Crystal data

**Table:**

[CdCl_2_(C_7_H_10_N_2_O_2_S)_2_]	*F*(000) = 2224
*M**_r_* = 555.76	*D*_x_ = 1.684 Mg m^−^^3^
Monoclinic, *C**c*	Mo *K*α radiation, λ = 0.71073 Å
Hall symbol: C -2yc	Cell parameters from 8288 reflections
*a* = 16.860 (3) Å	θ = 4.8–56.3°
*b* = 16.630 (3) Å	µ = 1.46 mm^−^^1^
*c* = 16.220 (3) Å	*T* = 293 K
β = 105.41 (3)°	Cube, yellow
*V* = 4384.3 (15) Å^3^	0.13 × 0.11 × 0.08 mm
*Z* = 8	

### Data collection

**Table:**

Bruker APEXII CCD area-detector diffractometer	8707 independent reflections
Radiation source: fine-focus sealed tube	8223 reflections with *I* > 2σ(*I*)
Graphite monochromator	*R*_int_ = 0.015
φ and ω scans	θ_max_ = 28.4°, θ_min_ = 1.8°
Absorption correction: multi-scan (*SADABS*; Sheldrick, 1996)	*h* = −16→22
*T*_min_ = 0.833, *T*_max_ = 0.892	*k* = −21→18
14070 measured reflections	*l* = −21→20

### Refinement

**Table:**

Refinement on *F*^2^	Secondary atom site location: difference Fourier map
Least-squares matrix: full	Hydrogen site location: inferred from neighbouring sites
*R*[*F*^2^ > 2σ(*F*^2^)] = 0.022	H-atom parameters constrained
*wR*(*F*^2^) = 0.048	*w* = 1/[σ^2^(*F*_o_^2^) + (0.0214*P*)^2^] where *P* = (*F*_o_^2^ + 2*F*_c_^2^)/3
*S* = 1.02	(Δ/σ)_max_ = 0.002
8707 reflections	Δρ_max_ = 0.48 e Å^−^^3^
492 parameters	Δρ_min_ = −0.28 e Å^−^^3^
2 restraints	Absolute structure: Flack (1983), 3396 Friedel pairs
Primary atom site location: structure-invariant direct methods	Flack parameter: 0.003 (12)

### Special details

**Table:**

Geometry. All esds (except the esd in the dihedral angle between two l.s. planes) are estimated using the full covariance matrix. The cell esds are taken into account individually in the estimation of esds in distances, angles and torsion angles; correlations between esds in cell parameters are only used when they are defined by crystal symmetry. An appro ximate (isotropic) treatment of cell esds is used for estimating esds involving l.s. planes.
Refinement. Refinement of F^2^ against ALL reflections. The weighted R-factor wR and goodness of fit S are based on F^2^, conventional R-factors R are based on F, with F set to zero for negative F^2^. The threshold expression of F^2^ > 2sigma(F^2^) is used only for calculating R-factors(gt) etc. and is not relevant to the choice of reflections for refinement. R-factors based on F^2^ are statistically about twice as large as those based on F, and R- factors based on ALL data will be even larger.

### Fractional atomic coordinates and isotropic or equivalent isotropic displacement parameters (Å^2^)

**Table:**

	*x*	*y*	*z*	*U*_iso_*/*U*_eq_	
Cd2	0.485132 (11)	−0.050594 (11)	0.483618 (12)	0.03730 (5)	
Cl1	0.38342 (5)	−0.15043 (5)	0.51743 (7)	0.0622 (2)	
Cl2	0.60420 (5)	−0.05382 (6)	0.61452 (6)	0.0573 (2)	
S1	0.32127 (6)	0.17849 (6)	0.52649 (8)	0.0677 (3)	
S2	0.57839 (7)	−0.27697 (5)	0.35146 (8)	0.0709 (3)	
O1	0.5970 (2)	0.17949 (17)	0.4071 (2)	0.0845 (9)	
O2	0.35439 (19)	0.00228 (19)	0.21198 (17)	0.0745 (7)	
O4	0.55304 (17)	0.05458 (14)	0.41459 (17)	0.0589 (6)	
O6	0.38851 (15)	−0.03458 (16)	0.34818 (16)	0.0607 (6)	
N1	0.42536 (14)	0.06974 (14)	0.51274 (15)	0.0370 (5)	
N2	0.53823 (15)	−0.13812 (15)	0.39801 (17)	0.0443 (6)	
N7	0.29128 (17)	0.02223 (16)	0.4987 (2)	0.0570 (8)	
H7A	0.3046	−0.0264	0.4902	0.068*	
H7B	0.2415	0.0334	0.4988	0.068*	
N8	0.60648 (19)	−0.22120 (17)	0.5118 (2)	0.0624 (8)	
H8A	0.6041	−0.1854	0.5493	0.075*	
H8B	0.6295	−0.2668	0.5279	0.075*	
C3	0.46569 (19)	0.14287 (17)	0.5250 (2)	0.0424 (7)	
C14	0.55607 (19)	0.14566 (19)	0.5285 (2)	0.0493 (8)	
H14A	0.5769	0.1998	0.5420	0.059*	
H14B	0.5865	0.1099	0.5730	0.059*	
C18	0.34717 (18)	0.07983 (17)	0.51152 (19)	0.0418 (6)	
C20	0.57463 (19)	−0.20604 (18)	0.4289 (2)	0.0493 (8)	
C23	0.56813 (19)	0.1206 (2)	0.4444 (2)	0.0504 (8)	
C31	0.4774 (2)	−0.0671 (2)	0.2587 (2)	0.0584 (9)	
H31A	0.5194	−0.0257	0.2681	0.070*	
H31B	0.4634	−0.0811	0.1985	0.070*	
C32	0.5135 (2)	−0.1403 (2)	0.3099 (2)	0.0504 (8)	
C33	0.4027 (2)	−0.0327 (2)	0.2788 (2)	0.0510 (8)	
C35	0.4212 (2)	0.2063 (2)	0.5344 (3)	0.0602 (9)	
H35A	0.4412	0.2586	0.5438	0.072*	
C36	0.5297 (2)	−0.2101 (3)	0.2750 (3)	0.0679 (11)	
H36A	0.5160	−0.2203	0.2165	0.081*	
C38	0.2991 (3)	0.1286 (4)	0.2447 (4)	0.1113 (19)	
H38A	0.2488	0.1565	0.2427	0.167*	
H38B	0.3248	0.1522	0.2043	0.167*	
H38C	0.3354	0.1327	0.3012	0.167*	
C39	0.2811 (3)	0.0433 (3)	0.2232 (3)	0.0775 (12)	
H39A	0.2629	0.0174	0.2686	0.093*	
H39B	0.2371	0.0394	0.1709	0.093*	
C41	0.6056 (4)	0.1653 (3)	0.3213 (4)	0.113 (2)	
H41A	0.6113	0.1082	0.3122	0.135*	
H41B	0.6543	0.1924	0.3141	0.135*	
C43	0.5325 (5)	0.1963 (5)	0.2599 (5)	0.155 (3)	
H43A	0.5421	0.1983	0.2042	0.233*	
H43B	0.5207	0.2494	0.2766	0.233*	
H43C	0.4867	0.1616	0.2585	0.233*	
Cd1	1.014298 (12)	−0.019131 (11)	0.516063 (12)	0.03457 (5)	
Cl3	1.05317 (5)	−0.12360 (5)	0.63094 (5)	0.04869 (18)	
Cl4	1.13217 (4)	0.08244 (4)	0.56401 (5)	0.04280 (16)	
S3	1.18288 (6)	−0.11604 (6)	0.34483 (6)	0.0617 (2)	
S4	0.80771 (6)	0.15640 (6)	0.57326 (9)	0.0819 (4)	
O3	0.88349 (16)	0.10310 (18)	0.27228 (15)	0.0719 (8)	
O5	0.76489 (15)	−0.14017 (18)	0.42157 (16)	0.0714 (7)	
O7	0.89008 (13)	−0.09262 (15)	0.43589 (14)	0.0526 (5)	
O8	0.95805 (15)	0.07188 (14)	0.40191 (15)	0.0559 (6)	
N3	0.90975 (15)	0.04584 (14)	0.56106 (17)	0.0395 (5)	
N4	1.06655 (14)	−0.07518 (14)	0.41083 (15)	0.0386 (5)	
N5	1.17737 (16)	−0.13937 (18)	0.50584 (18)	0.0533 (7)	
H5A	1.1551	−0.1358	0.5475	0.064*	
H5B	1.2248	−0.1618	0.5135	0.064*	
N6	0.96308 (16)	0.17675 (17)	0.5695 (2)	0.0617 (8)	
H6A	1.0110	0.1603	0.5678	0.074*	
H6B	0.9540	0.2274	0.5731	0.074*	
C7	0.90311 (18)	0.12415 (19)	0.5664 (2)	0.0454 (7)	
C15	0.83681 (18)	0.0086 (2)	0.5619 (2)	0.0449 (7)	
C16	1.13877 (18)	−0.11042 (18)	0.4296 (2)	0.0425 (7)	
C19	0.83232 (19)	−0.10455 (19)	0.4647 (2)	0.0476 (7)	
C24	1.0442 (2)	−0.0512 (2)	0.3255 (2)	0.0469 (7)	
C25	0.9363 (2)	0.0568 (2)	0.3274 (2)	0.0495 (8)	
C27	0.9611 (2)	−0.0157 (2)	0.2847 (2)	0.0574 (8)	
H27A	0.9201	−0.0572	0.2818	0.069*	
H27B	0.9595	−0.0011	0.2263	0.069*	
C29	0.8293 (2)	−0.0817 (2)	0.5528 (2)	0.0498 (8)	
H29A	0.8739	−0.1074	0.5947	0.060*	
H29B	0.7777	−0.0993	0.5626	0.060*	
C30	1.0989 (2)	−0.0682 (2)	0.2821 (2)	0.0599 (9)	
H30A	1.0922	−0.0556	0.2247	0.072*	
C34	0.7765 (2)	0.0577 (2)	0.5684 (3)	0.0680 (11)	
H34A	0.7244	0.0408	0.5701	0.082*	
C37	0.8494 (3)	0.1722 (3)	0.3080 (3)	0.0815 (13)	
H37A	0.8166	0.1538	0.3452	0.098*	
H37B	0.8935	0.2057	0.3412	0.098*	
C40	0.7588 (3)	−0.1582 (4)	0.3317 (3)	0.121 (2)	
H40A	0.8131	−0.1707	0.3254	0.146*	
H40B	0.7243	−0.2051	0.3144	0.146*	
C42	0.7984 (4)	0.2179 (4)	0.2368 (3)	0.120 (2)	
H42A	0.7847	0.2689	0.2571	0.179*	
H42B	0.7489	0.1885	0.2118	0.179*	
H42C	0.8281	0.2264	0.1946	0.179*	
C44	0.7253 (4)	−0.0925 (8)	0.2773 (5)	0.194 (5)	
H44A	0.7089	−0.1105	0.2190	0.291*	
H44B	0.7661	−0.0510	0.2832	0.291*	
H44C	0.6783	−0.0715	0.2930	0.291*	

### Atomic displacement parameters (Å^2^)

**Table:**

	*U*^11^	*U*^22^	*U*^33^	*U*^12^	*U*^13^	*U*^23^
Cd2	0.03566 (10)	0.03306 (10)	0.04408 (11)	0.00243 (9)	0.01218 (8)	0.00147 (9)
Cl1	0.0430 (4)	0.0399 (4)	0.1096 (7)	0.0052 (3)	0.0305 (4)	0.0189 (4)
Cl2	0.0486 (4)	0.0598 (5)	0.0562 (5)	−0.0012 (4)	0.0010 (4)	0.0068 (4)
S1	0.0590 (5)	0.0406 (5)	0.1071 (8)	0.0101 (4)	0.0285 (5)	−0.0137 (5)
S2	0.0710 (6)	0.0411 (5)	0.1128 (8)	−0.0020 (4)	0.0456 (6)	−0.0174 (5)
O1	0.121 (2)	0.0509 (15)	0.103 (2)	−0.0169 (16)	0.066 (2)	0.0038 (15)
O2	0.0798 (18)	0.085 (2)	0.0513 (15)	0.0108 (15)	0.0053 (13)	0.0091 (13)
O4	0.0739 (17)	0.0446 (14)	0.0666 (16)	−0.0107 (12)	0.0334 (13)	−0.0010 (11)
O6	0.0545 (14)	0.0800 (18)	0.0478 (14)	0.0151 (12)	0.0138 (11)	0.0068 (12)
N1	0.0365 (12)	0.0302 (12)	0.0459 (13)	0.0005 (10)	0.0135 (10)	−0.0027 (10)
N2	0.0436 (13)	0.0367 (13)	0.0577 (16)	0.0005 (11)	0.0225 (12)	−0.0011 (11)
N7	0.0408 (15)	0.0421 (15)	0.095 (2)	0.0009 (12)	0.0307 (15)	−0.0132 (15)
N8	0.0664 (18)	0.0423 (16)	0.082 (2)	0.0204 (14)	0.0251 (16)	0.0147 (15)
C3	0.0473 (16)	0.0349 (16)	0.0437 (16)	−0.0024 (13)	0.0097 (13)	−0.0022 (12)
C14	0.0442 (17)	0.0398 (17)	0.062 (2)	−0.0096 (14)	0.0107 (14)	−0.0028 (14)
C18	0.0445 (16)	0.0342 (15)	0.0489 (17)	0.0031 (13)	0.0163 (13)	−0.0032 (12)
C20	0.0430 (16)	0.0352 (16)	0.079 (2)	0.0018 (13)	0.0317 (16)	0.0010 (15)
C23	0.0409 (16)	0.0473 (19)	0.065 (2)	−0.0041 (14)	0.0183 (14)	0.0073 (16)
C31	0.068 (2)	0.061 (2)	0.0495 (19)	−0.0025 (18)	0.0224 (17)	−0.0046 (16)
C32	0.0482 (17)	0.0515 (19)	0.056 (2)	−0.0060 (15)	0.0213 (15)	−0.0058 (15)
C33	0.055 (2)	0.0463 (18)	0.0482 (19)	−0.0081 (14)	0.0075 (15)	−0.0026 (14)
C35	0.060 (2)	0.0325 (17)	0.089 (3)	−0.0015 (15)	0.0204 (19)	−0.0082 (16)
C36	0.067 (2)	0.068 (3)	0.076 (3)	−0.0113 (19)	0.032 (2)	−0.024 (2)
C38	0.092 (4)	0.094 (4)	0.144 (5)	0.006 (3)	0.023 (4)	−0.015 (4)
C39	0.055 (2)	0.087 (3)	0.080 (3)	0.007 (2)	−0.001 (2)	0.018 (2)
C41	0.163 (6)	0.089 (4)	0.119 (5)	−0.024 (4)	0.093 (5)	0.010 (3)
C43	0.188 (8)	0.182 (8)	0.095 (5)	0.020 (7)	0.038 (5)	−0.035 (5)
Cd1	0.03415 (9)	0.03501 (10)	0.03617 (9)	0.00204 (9)	0.01218 (7)	0.00289 (8)
Cl3	0.0538 (4)	0.0426 (4)	0.0497 (4)	−0.0013 (3)	0.0139 (3)	0.0144 (3)
Cl4	0.0347 (3)	0.0369 (4)	0.0590 (4)	0.0000 (3)	0.0164 (3)	0.0013 (3)
S3	0.0545 (5)	0.0756 (6)	0.0644 (5)	0.0017 (4)	0.0322 (4)	−0.0149 (5)
S4	0.0514 (5)	0.0565 (6)	0.1508 (11)	0.0117 (4)	0.0493 (6)	−0.0076 (6)
O3	0.0619 (15)	0.101 (2)	0.0495 (14)	0.0320 (15)	0.0092 (12)	0.0130 (14)
O5	0.0548 (15)	0.090 (2)	0.0695 (17)	−0.0322 (14)	0.0169 (12)	−0.0130 (14)
O7	0.0440 (12)	0.0659 (15)	0.0514 (12)	−0.0130 (11)	0.0186 (10)	−0.0070 (11)
O8	0.0655 (15)	0.0555 (14)	0.0442 (13)	0.0079 (11)	0.0100 (11)	0.0085 (10)
N3	0.0358 (13)	0.0365 (13)	0.0493 (14)	0.0029 (10)	0.0169 (11)	0.0026 (10)
N4	0.0382 (13)	0.0406 (13)	0.0398 (13)	−0.0041 (10)	0.0153 (10)	−0.0046 (10)
N5	0.0414 (14)	0.0585 (18)	0.0611 (17)	0.0124 (13)	0.0154 (13)	0.0036 (14)
N6	0.0426 (15)	0.0356 (14)	0.113 (3)	0.0029 (12)	0.0303 (16)	−0.0039 (15)
C7	0.0370 (15)	0.0416 (17)	0.0596 (19)	0.0053 (13)	0.0166 (14)	−0.0016 (14)
C15	0.0378 (15)	0.0477 (18)	0.0529 (18)	−0.0026 (13)	0.0186 (13)	−0.0003 (14)
C16	0.0378 (15)	0.0375 (16)	0.0547 (18)	−0.0035 (12)	0.0165 (13)	−0.0075 (13)
C19	0.0414 (16)	0.0433 (17)	0.0558 (19)	−0.0070 (13)	0.0087 (14)	0.0029 (14)
C24	0.0487 (17)	0.0526 (18)	0.0400 (16)	−0.0064 (14)	0.0126 (13)	−0.0047 (13)
C25	0.0437 (17)	0.060 (2)	0.0475 (19)	0.0031 (15)	0.0170 (14)	0.0151 (15)
C27	0.0554 (19)	0.074 (2)	0.0390 (17)	0.0023 (18)	0.0054 (14)	0.0020 (16)
C29	0.0453 (17)	0.0503 (19)	0.0578 (19)	−0.0073 (15)	0.0208 (15)	0.0054 (15)
C30	0.064 (2)	0.072 (2)	0.049 (2)	−0.0088 (19)	0.0260 (17)	−0.0085 (17)
C34	0.0437 (19)	0.061 (2)	0.108 (3)	0.0000 (17)	0.037 (2)	−0.003 (2)
C37	0.074 (3)	0.102 (3)	0.070 (3)	0.037 (3)	0.021 (2)	0.014 (2)
C40	0.082 (3)	0.199 (7)	0.083 (3)	−0.081 (4)	0.023 (3)	−0.041 (4)
C42	0.127 (5)	0.144 (6)	0.096 (4)	0.063 (4)	0.044 (3)	0.027 (4)
C44	0.086 (4)	0.386 (16)	0.101 (5)	−0.010 (6)	0.011 (4)	0.101 (8)

### Geometric parameters (Å, º)

**Table:**

Cd1—N3	2.343 (2)	C41—C43	1.457 (9)
Cd1—N4	2.315 (2)	C41—H41A	0.9700
Cd1—O7	2.475 (2)	C41—H41B	0.9700
Cd1—O8	2.384 (2)	C43—H43A	0.9600
Cd1—Cl3	2.5041 (8)	C43—H43B	0.9600
Cd1—Cl4	2.5664 (8)	C43—H43C	0.9600
Cd2—N1	2.344 (2)	S3—C30	1.705 (4)
Cd2—N2	2.347 (2)	S3—C16	1.730 (3)
Cd2—O4	2.511 (2)	S4—C34	1.720 (4)
Cd2—O6	2.377 (3)	S4—C7	1.727 (3)
Cd2—Cl1	2.5491 (9)	O3—C25	1.327 (4)
Cd2—Cl2	2.5051 (12)	O3—C37	1.472 (5)
S1—C35	1.719 (4)	O5—C19	1.307 (4)
S1—C18	1.731 (3)	O5—C40	1.465 (6)
S2—C36	1.706 (5)	O7—C19	1.203 (4)
S2—C20	1.736 (3)	O8—C25	1.193 (4)
O1—C23	1.310 (4)	N3—C7	1.312 (4)
O1—C41	1.456 (6)	N3—C15	1.380 (4)
O2—C33	1.307 (4)	N4—C16	1.312 (4)
O2—C39	1.465 (5)	N4—C24	1.393 (4)
O4—C23	1.200 (4)	N5—C16	1.326 (4)
O6—C33	1.212 (4)	N5—H5A	0.8579
N1—C18	1.324 (4)	N5—H5B	0.8610
N1—C3	1.382 (4)	N6—C7	1.328 (4)
N2—C20	1.319 (4)	N6—H6A	0.8603
N2—C32	1.379 (4)	N6—H6B	0.8609
N7—C18	1.321 (4)	C15—C34	1.330 (5)
N7—H7A	0.8602	C15—C29	1.510 (5)
N7—H7B	0.8600	C19—C29	1.493 (5)
N8—C20	1.334 (5)	C24—C30	1.332 (5)
N8—H8A	0.8596	C24—C27	1.501 (5)
N8—H8B	0.8602	C25—C27	1.504 (5)
C3—C35	1.327 (4)	C27—H27A	0.9700
C3—C14	1.511 (4)	C27—H27B	0.9700
C14—C23	1.491 (5)	C29—H29A	0.9700
C14—H14A	0.9700	C29—H29B	0.9700
C14—H14B	0.9700	C30—H30A	0.9300
C31—C33	1.496 (5)	C34—H34A	0.9300
C31—C32	1.507 (5)	C37—C42	1.457 (6)
C31—H31A	0.9700	C37—H37A	0.9700
C31—H31B	0.9700	C37—H37B	0.9700
C32—C36	1.350 (5)	C40—C44	1.423 (10)
C35—H35A	0.9300	C40—H40A	0.9700
C36—H36A	0.9300	C40—H40B	0.9700
C38—C39	1.473 (7)	C42—H42A	0.9600
C38—H38A	0.9600	C42—H42B	0.9600
C38—H38B	0.9600	C42—H42C	0.9600
C38—H38C	0.9600	C44—H44A	0.9600
C39—H39A	0.9700	C44—H44B	0.9600
C39—H39B	0.9700	C44—H44C	0.9600
			
N1—Cd2—N2	154.22 (9)	N4—Cd1—N3	151.71 (9)
N1—Cd2—O6	82.23 (8)	N4—Cd1—O8	80.43 (8)
N2—Cd2—O6	78.35 (9)	N3—Cd1—O8	76.84 (9)
N1—Cd2—Cl2	97.94 (6)	N4—Cd1—O7	81.28 (8)
N2—Cd2—Cl2	98.19 (7)	N3—Cd1—O7	77.87 (8)
O6—Cd2—Cl2	169.70 (7)	O8—Cd1—O7	78.40 (9)
N1—Cd2—O4	76.50 (8)	N4—Cd1—Cl3	101.17 (7)
N2—Cd2—O4	82.96 (8)	N3—Cd1—Cl3	99.09 (7)
O6—Cd2—O4	77.97 (9)	O8—Cd1—Cl3	171.75 (6)
Cl2—Cd2—O4	92.02 (7)	O7—Cd1—Cl3	93.79 (6)
N1—Cd2—Cl1	99.37 (6)	N4—Cd1—Cl4	94.17 (6)
N2—Cd2—Cl1	96.17 (7)	N3—Cd1—Cl4	100.84 (6)
O6—Cd2—Cl1	86.37 (7)	O8—Cd1—Cl4	86.20 (7)
Cl2—Cd2—Cl1	103.71 (3)	O7—Cd1—Cl4	164.45 (6)
O4—Cd2—Cl1	164.19 (7)	Cl3—Cd1—Cl4	101.69 (3)
C35—S1—C18	89.26 (15)	C30—S3—C16	89.15 (16)
C36—S2—C20	88.85 (18)	C34—S4—C7	88.83 (17)
C23—O1—C41	117.6 (3)	C25—O3—C37	116.7 (3)
C33—O2—C39	117.7 (3)	C19—O5—C40	116.5 (3)
C23—O4—Cd2	122.1 (2)	C19—O7—Cd1	122.5 (2)
C33—O6—Cd2	127.2 (2)	C25—O8—Cd1	127.3 (2)
C18—N1—C3	109.9 (2)	C7—N3—C15	110.7 (3)
C18—N1—Cd2	125.78 (19)	C7—N3—Cd1	124.3 (2)
C3—N1—Cd2	123.98 (18)	C15—N3—Cd1	122.8 (2)
C20—N2—C32	110.7 (3)	C16—N4—C24	110.4 (3)
C20—N2—Cd2	121.2 (2)	C16—N4—Cd1	121.4 (2)
C32—N2—Cd2	124.9 (2)	C24—N4—Cd1	124.2 (2)
C18—N7—H7A	120.1	C16—N5—H5A	120.1
C18—N7—H7B	119.9	C16—N5—H5B	119.9
H7A—N7—H7B	120.0	H5A—N5—H5B	120.0
C20—N8—H8A	120.1	C7—N6—H6A	120.1
C20—N8—H8B	120.1	C7—N6—H6B	120.0
H8A—N8—H8B	119.9	H6A—N6—H6B	119.9
C35—C3—N1	116.4 (3)	N3—C7—N6	125.4 (3)
C35—C3—C14	124.5 (3)	N3—C7—S4	114.2 (2)
N1—C3—C14	119.0 (3)	N6—C7—S4	120.4 (2)
C23—C14—C3	109.8 (3)	C34—C15—N3	115.3 (3)
C23—C14—H14A	109.7	C34—C15—C29	124.9 (3)
C3—C14—H14A	109.7	N3—C15—C29	119.8 (3)
C23—C14—H14B	109.7	N4—C16—N5	125.0 (3)
C3—C14—H14B	109.7	N4—C16—S3	114.1 (2)
H14A—C14—H14B	108.2	N5—C16—S3	120.9 (2)
N7—C18—N1	125.2 (3)	O7—C19—O5	123.1 (3)
N7—C18—S1	120.9 (2)	O7—C19—C29	124.5 (3)
N1—C18—S1	113.9 (2)	O5—C19—C29	112.4 (3)
N2—C20—N8	124.5 (3)	C30—C24—N4	114.9 (3)
N2—C20—S2	114.2 (3)	C30—C24—C27	123.1 (3)
N8—C20—S2	121.3 (2)	N4—C24—C27	121.7 (3)
O4—C23—O1	124.3 (3)	O8—C25—O3	122.1 (3)
O4—C23—C14	123.9 (3)	O8—C25—C27	125.9 (3)
O1—C23—C14	111.8 (3)	O3—C25—C27	111.9 (3)
C33—C31—C32	115.4 (3)	C24—C27—C25	116.9 (3)
C33—C31—H31A	108.4	C24—C27—H27A	108.1
C32—C31—H31A	108.4	C25—C27—H27A	108.1
C33—C31—H31B	108.4	C24—C27—H27B	108.1
C32—C31—H31B	108.4	C25—C27—H27B	108.1
H31A—C31—H31B	107.5	H27A—C27—H27B	107.3
C36—C32—N2	114.7 (3)	C19—C29—C15	108.9 (3)
C36—C32—C31	124.0 (3)	C19—C29—H29A	109.9
N2—C32—C31	121.1 (3)	C15—C29—H29A	109.9
O6—C33—O2	123.2 (3)	C19—C29—H29B	109.9
O6—C33—C31	125.3 (3)	C15—C29—H29B	109.9
O2—C33—C31	111.5 (3)	H29A—C29—H29B	108.3
C3—C35—S1	110.5 (2)	C24—C30—S3	111.4 (3)
C3—C35—H35A	124.8	C24—C30—H30A	124.3
S1—C35—H35A	124.8	S3—C30—H30A	124.3
C32—C36—S2	111.5 (3)	C15—C34—S4	111.0 (3)
C32—C36—H36A	124.2	C15—C34—H34A	124.5
S2—C36—H36A	124.2	S4—C34—H34A	124.5
C39—C38—H38A	109.5	C42—C37—O3	107.8 (4)
C39—C38—H38B	109.5	C42—C37—H37A	110.2
H38A—C38—H38B	109.5	O3—C37—H37A	110.2
C39—C38—H38C	109.5	C42—C37—H37B	110.2
H38A—C38—H38C	109.5	O3—C37—H37B	110.2
H38B—C38—H38C	109.5	H37A—C37—H37B	108.5
O2—C39—C38	110.5 (4)	C44—C40—O5	111.8 (7)
O2—C39—H39A	109.5	C44—C40—H40A	109.3
C38—C39—H39A	109.5	O5—C40—H40A	109.3
O2—C39—H39B	109.5	C44—C40—H40B	109.3
C38—C39—H39B	109.5	O5—C40—H40B	109.3
H39A—C39—H39B	108.1	H40A—C40—H40B	107.9
O1—C41—C43	108.4 (5)	C37—C42—H42A	109.5
O1—C41—H41A	110.0	C37—C42—H42B	109.5
C43—C41—H41A	110.0	H42A—C42—H42B	109.5
O1—C41—H41B	110.0	C37—C42—H42C	109.5
C43—C41—H41B	110.0	H42A—C42—H42C	109.5
H41A—C41—H41B	108.4	H42B—C42—H42C	109.5
C41—C43—H43A	109.5	C40—C44—H44A	109.5
C41—C43—H43B	109.5	C40—C44—H44B	109.5
H43A—C43—H43B	109.5	H44A—C44—H44B	109.5
C41—C43—H43C	109.5	C40—C44—H44C	109.5
H43A—C43—H43C	109.5	H44A—C44—H44C	109.5
H43B—C43—H43C	109.5	H44B—C44—H44C	109.5
			
N1—Cd2—O4—C23	34.1 (3)	N4—Cd1—O7—C19	165.4 (3)
N2—Cd2—O4—C23	−161.6 (3)	N3—Cd1—O7—C19	−33.8 (3)
O6—Cd2—O4—C23	118.9 (3)	O8—Cd1—O7—C19	−112.6 (3)
Cl2—Cd2—O4—C23	−63.6 (3)	Cl3—Cd1—O7—C19	64.7 (3)
Cl1—Cd2—O4—C23	110.6 (3)	Cl4—Cd1—O7—C19	−120.6 (3)
N1—Cd2—O6—C33	124.6 (3)	N4—Cd1—O8—C25	33.3 (3)
N2—Cd2—O6—C33	−38.3 (3)	N3—Cd1—O8—C25	−129.8 (3)
Cl2—Cd2—O6—C33	33.0 (6)	O7—Cd1—O8—C25	−49.7 (3)
O4—Cd2—O6—C33	46.9 (3)	Cl4—Cd1—O8—C25	128.2 (3)
Cl1—Cd2—O6—C33	−135.4 (3)	N4—Cd1—N3—C7	−91.3 (3)
N2—Cd2—N1—C18	102.8 (3)	O8—Cd1—N3—C7	−53.9 (3)
O6—Cd2—N1—C18	61.4 (2)	O7—Cd1—N3—C7	−134.7 (3)
Cl2—Cd2—N1—C18	−129.0 (2)	Cl3—Cd1—N3—C7	133.4 (3)
O4—Cd2—N1—C18	140.9 (3)	Cl4—Cd1—N3—C7	29.5 (3)
Cl1—Cd2—N1—C18	−23.6 (2)	N4—Cd1—N3—C15	70.0 (3)
N2—Cd2—N1—C3	−70.1 (3)	O8—Cd1—N3—C15	107.4 (2)
O6—Cd2—N1—C3	−111.4 (2)	O7—Cd1—N3—C15	26.7 (2)
Cl2—Cd2—N1—C3	58.2 (2)	Cl3—Cd1—N3—C15	−65.3 (2)
O4—Cd2—N1—C3	−32.0 (2)	Cl4—Cd1—N3—C15	−169.2 (2)
Cl1—Cd2—N1—C3	163.6 (2)	N3—Cd1—N4—C16	−176.6 (2)
N1—Cd2—N2—C20	173.0 (2)	O8—Cd1—N4—C16	146.6 (2)
O6—Cd2—N2—C20	−145.1 (2)	O7—Cd1—N4—C16	−133.8 (2)
Cl2—Cd2—N2—C20	44.7 (2)	Cl3—Cd1—N4—C16	−41.6 (2)
O4—Cd2—N2—C20	135.8 (2)	Cl4—Cd1—N4—C16	61.2 (2)
Cl1—Cd2—N2—C20	−60.1 (2)	N3—Cd1—N4—C24	28.4 (3)
N1—Cd2—N2—C32	−29.3 (4)	O8—Cd1—N4—C24	−8.4 (2)
O6—Cd2—N2—C32	12.6 (2)	O7—Cd1—N4—C24	71.2 (2)
Cl2—Cd2—N2—C32	−157.5 (2)	Cl3—Cd1—N4—C24	163.4 (2)
O4—Cd2—N2—C32	−66.5 (2)	Cl4—Cd1—N4—C24	−93.8 (2)
Cl1—Cd2—N2—C32	97.6 (2)	C15—N3—C7—N6	178.1 (3)
C18—N1—C3—C35	1.1 (4)	Cd1—N3—C7—N6	−18.6 (5)
Cd2—N1—C3—C35	175.0 (2)	C15—N3—C7—S4	0.3 (4)
C18—N1—C3—C14	−179.9 (3)	Cd1—N3—C7—S4	163.56 (15)
Cd2—N1—C3—C14	−6.1 (4)	C34—S4—C7—N3	0.0 (3)
C35—C3—C14—C23	−117.1 (4)	C34—S4—C7—N6	−178.0 (3)
N1—C3—C14—C23	64.0 (4)	C7—N3—C15—C34	−0.5 (4)
C3—N1—C18—N7	178.1 (3)	Cd1—N3—C15—C34	−164.0 (3)
Cd2—N1—C18—N7	4.3 (5)	C7—N3—C15—C29	177.6 (3)
C3—N1—C18—S1	−0.7 (3)	Cd1—N3—C15—C29	14.0 (4)
Cd2—N1—C18—S1	−174.40 (13)	C24—N4—C16—N5	179.4 (3)
C35—S1—C18—N7	−178.7 (3)	Cd1—N4—C16—N5	21.3 (4)
C35—S1—C18—N1	0.1 (3)	C24—N4—C16—S3	0.0 (3)
C32—N2—C20—N8	178.7 (3)	Cd1—N4—C16—S3	−158.08 (14)
Cd2—N2—C20—N8	−20.7 (4)	C30—S3—C16—N4	0.0 (3)
C32—N2—C20—S2	−1.3 (3)	C30—S3—C16—N5	−179.3 (3)
Cd2—N2—C20—S2	159.31 (14)	Cd1—O7—C19—O5	176.9 (2)
C36—S2—C20—N2	0.8 (3)	Cd1—O7—C19—C29	−3.8 (4)
C36—S2—C20—N8	−179.2 (3)	C40—O5—C19—O7	−6.2 (6)
Cd2—O4—C23—O1	−174.0 (3)	C40—O5—C19—C29	174.4 (4)
Cd2—O4—C23—C14	5.9 (5)	C16—N4—C24—C30	−0.1 (4)
C41—O1—C23—O4	4.6 (6)	Cd1—N4—C24—C30	157.3 (2)
C41—O1—C23—C14	−175.3 (4)	C16—N4—C24—C27	174.6 (3)
C3—C14—C23—O4	−64.5 (5)	Cd1—N4—C24—C27	−28.0 (4)
C3—C14—C23—O1	115.4 (3)	Cd1—O8—C25—O3	158.4 (2)
C20—N2—C32—C36	1.3 (4)	Cd1—O8—C25—C27	−19.4 (5)
Cd2—N2—C32—C36	−158.5 (2)	C37—O3—C25—O8	−3.1 (5)
C20—N2—C32—C31	−174.2 (3)	C37—O3—C25—C27	175.0 (3)
Cd2—N2—C32—C31	26.1 (4)	C30—C24—C27—C25	−130.4 (4)
C33—C31—C32—C36	127.5 (4)	N4—C24—C27—C25	55.3 (5)
C33—C31—C32—N2	−57.5 (4)	O8—C25—C27—C24	−29.2 (5)
Cd2—O6—C33—O2	−155.9 (3)	O3—C25—C27—C24	152.9 (3)
Cd2—O6—C33—C31	22.2 (5)	O7—C19—C29—C15	62.1 (4)
C39—O2—C33—O6	2.2 (5)	O5—C19—C29—C15	−118.6 (3)
C39—O2—C33—C31	−176.2 (3)	C34—C15—C29—C19	110.3 (4)
C32—C31—C33—O6	31.3 (5)	N3—C15—C29—C19	−67.5 (4)
C32—C31—C33—O2	−150.3 (3)	N4—C24—C30—S3	0.1 (4)
N1—C3—C35—S1	−1.0 (4)	C27—C24—C30—S3	−174.5 (3)
C14—C3—C35—S1	−179.9 (3)	C16—S3—C30—C24	−0.1 (3)
C18—S1—C35—C3	0.5 (3)	N3—C15—C34—S4	0.5 (4)
N2—C32—C36—S2	−0.7 (4)	C29—C15—C34—S4	−177.5 (3)
C31—C32—C36—S2	174.6 (3)	C7—S4—C34—C15	−0.2 (3)
C20—S2—C36—C32	−0.1 (3)	C25—O3—C37—C42	175.8 (4)
C33—O2—C39—C38	91.7 (5)	C19—O5—C40—C44	−87.5 (5)
C23—O1—C41—C43	95.7 (6)		

### Hydrogen-bond geometry (Å, º)

**Table:**

*D*—H···*A*	*D*—H	H···*A*	*D*···*A*	*D*—H···*A*
N5—H5*A*···Cl3	0.86	2.46	3.291 (3)	163
N6—H6*A*···Cl4	0.86	2.43	3.277 (3)	167
N7—H7*A*···Cl1	0.86	2.43	3.242 (3)	157
N8—H8*A*···Cl2	0.86	2.43	3.248 (3)	160
C14—H14*B*···Cl2	0.97	2.80	3.607 (3)	141
C34—H34*A*···Cl2	0.93	2.81	3.687 (4)	158
N5—H5*B*···Cl1^i^	0.86	2.67	3.435 (3)	149
N6—H6*B*···Cl1^ii^	0.86	2.41	3.189 (3)	152
N8—H8*B*···Cl4^iii^	0.86	2.57	3.373 (3)	155
C35—H35*A*···Cl3^iv^	0.93	2.82	3.681 (4)	153

Symmetry codes: (i) *x*+1, *y*, *z*; (ii) *x*+1/2, *y*+1/2, *z*; (iii) *x*−1/2, *y*−1/2, *z*; (iv) *x*−1/2, *y*+1/2, *z*.
